# Efficacy of walking as a potential strategy to treat childhood obesity in the clinical setting

**DOI:** 10.1111/ped.70120

**Published:** 2025-06-04

**Authors:** Kiwako Miura, Yumiko Ninomiya, Sachie Sakimukai, Yoshiya Ito, Masao Yoshinaga

**Affiliations:** ^1^ Department of Pediatrics National Hospital Organization (NHO) Kagoshima Medical Center Kagoshima Japan; ^2^ Nutritional Management Room National Hospital Organization (NHO) Kagoshima Medical Center Kagoshima Japan; ^3^ Division of Clinical Medicine Japanese Red Cross Hokkaido College of Nursing Kitami Hokkaido Japan

**Keywords:** children, light‐intensity physical activity, obesity treatment, relative body weight, walking

## Abstract

**Background:**

Walking is a common intervention for treating obesity in adults, but data on the effectiveness of walking for childhood obesity are limited. We therefore investigated the effectiveness of walking in the treatment of childhood obesity and the factors that make its effect stronger.

**Methods:**

Participants who visited our clinic for obesity were instructed to walk at least 10,000 steps on holidays and given lifestyle guidance at the first visit. CV risk factors and blood chemistry were examined at every visit. The number of steps walked on holidays between each visit was also assessed. We defined successful treatment as a final decrease in relative body weight (RBW) of ≥8.6% in this study. The predictors of final RBW reduction and factors of dropout were examined with a focus on the number of holiday steps.

**Results:**

The final number of participants was 131 (74 boys and 57 girls; mean age 10.1 ± 2.4 years). The mean reduction in RBW was 14.7 ± 12.8% (*p* < 0.001). Predictors of final RBW reduction were the level of RBW reduction from the first to the second visits (*p* = 0.01) and the mean number of steps on holiday between the second and the third visits (*p* = 0.04). Fewer steps on holiday between the first and the second visits were a predictor of dropout (*p* = 0.03).

**Conclusions:**

This study confirmed the effectiveness of lifestyle modification, particularly walking. Furthermore, the establishment of walking habits and reduction in RBW early in the treatment were found to be important.

## INTRODUCTION

The prevalence of childhood obesity has increased worldwide over the past five decades.^1^ From 1975 to 2016, the worldwide prevalence of obesity in children and adolescents aged 5–19 years increased from 0.8% to 5.7% for girls and from 0.9% to 7.8% for boys.^2^ The prevalence of childhood obesity in Japan peaked around 2000 at 8%–10% for girls and 10%–12% for boys and remained stable over the last decade, but has been on the rise again in recent years.^3^ A new concern is that the prevalence of childhood/adult obesity is expected to rise as a result of the COVID‐19 stay‐at‐home orders.^4^ An important issue in Japan is an abrupt increase in the prevalence of obesity during elementary school years.^5^ Obesity during childhood is likely to continue into adulthood and is associated with cardiometabolic and psychosocial comorbidity as well as premature mortality.^1^, ^6^, ^7^, ^8^ Then, prevention and treatment of childhood obesity are still a major concern worldwide.

Common strategies for preventing or treating obesity in children and adolescents are increasing physical activity (PA), modifying dietary intake, reducing sedentary behaviors, improving sleep patterns, providing parental and social support, and a combination of these strategies.^9^, ^10^ Daily moderate‐to‐vigorous PA (MVPA) for at least 60 min has been recommended in many countries for children and adolescents.^11^ However, surveys of global PA levels have reported that as many as 80% of 13‐ to 15‐year‐olds engage in less than 60 min of MVPA per day.^12^ Consequently, replacing MVPA with light‐intensity PA has been introduced to promote health and prevent cardiovascular (CV) diseases.^13^ Walking is a recommended activity for increasing the energy expenditure of obese individuals and has been used as an interventional arm for treating obesity in adults.^14^, ^15^ However, there are limited data on the effectiveness of walking for treating childhood obesity or improving CV risk levels in children with obesity.^16^

A general decrease in PA and an increase in sedentary lifestyle is a concern in Japanese children, particularly on school holidays, and this is exaggerated in children with obesity.^17^ One‐fourth of children decrease the number of steps by more than 50% on school holidays compared with school days (weekdays).^17^ A recent randomized controlled study in children showed that walking on school holidays is an effective way to treat obesity and to improve many CV risk factor levels in children with mild to moderate obesity.^18^ Our hypothesis was that promoting PA through walking on school holidays was an additional way to treat childhood obesity in children with obesity also in the clinical setting.

The aim of the present study was therefore to determine the efficacy of walking as an obesity treatment strategy for children and adolescents in the clinical setting. We also analyzed clinical factors in the early stages of treatment that affect the final treatment effect.

## METHODS

Participants were consecutive 171 children and adolescents aged ≤18 years (101 boys and 70 girls; mean age ± standard deviation, 10.1 ± 2.5 years; range, 5–18 years) who visited an outpatient clinic for obesity from April 2005 to December 2021 (Table 1). A retrospective statistical analysis was conducted on the basis of medical records. The inclusion criterion was participants who had visited the clinic four or more times to analyze the factors important for successful treatment during the early stage of treatment. Participants who did not fulfill these conditions were considered dropout cases. Participants were mainly followed by one pediatric physician (M.Y.) who had been certified by the Japan Society for the Study of Obesity. This study was approved by the Ethics Committee of the National Hospital Organization (NHO) Kagoshima Medical Center (No. 2021‐20).

**TABLE 1:** Baseline characteristics of participants.

	All	Continue	Dropout	*p* Value
Number of participants	171	131	40	
Age (years)	10.1 ± 2.5	10.1 ± 2.4	10.4 ± 2.8	0.60
Height (cm)	141.0 ± 13.3	140.6 ± 12.5	142.2 ± 15.8	0.50
Weight (kg)	53.1 ± 17.6	52.3 ± 16.3	55.5 ± 21.3	0.32
Relative body weight (%)	46.2 ± 20.3	45.8 ± 20.4	47.7 ± 19.8	0.59
Body mass index	26.0 ± 4.4	25.8 ± 4.2	26.4 ± 5.0	0.51
Body mass index‐Z scores	2.2 ± 0.5	2.2 ± 0.4	2.2 ± 0.5	0.68
Waist circumference (cm)	84.0 ± 12.1	83.9 ± 11.7	84.2 ± 13.5	0.88
Systolic blood pressure (mmHg)	108 ± 10	108 ± 10	108 ± 11	0.95
Diastolic blood pressure (mmHg)	62 ± 8	62 ± 8	61 ± 9	0.25
Aspartate aminotransferase (U/L)	31 ± 19	31 ± 19	31 ± 17	0.98
Alanine aminotransferase (U/L)	43 ± 48	42 ± 50	44 ± 42	0.78
Uric acid (mmol/L)	318.2 ± 77.2	318.6 ± 79.7	316.0 ± 68.5	0.86
Total cholesterol (mmol/L)	4.58 ± 0.79	4.61 ± 0.80	4.34 ± 0.78	0.06
Triglycerides (mmol/L)	1.07 ± 0.50	1.08 ± 0.50	1.04 ± 0.54	0.70
HDL‐cholesterol (mmol/L)	1.29 ± 0.27	1.29 ± 0.27	1.32 ± 0.26	0.54
LDL‐cholesterol (mmol/L)	2.87 ± 0.75	2.89 ± 0.75	2.67 ± 0.62	0.11
Fasting plasma glucose (mmol/L)	4.89 ± 0.46	4.88 ± 0.46	5.08 ± 0.48	0.02
Insulin (pmol/L)	122.3 ± 105.9	114.4 ± 63.5	137.1 ± 94.6	0.09
Father's age (year)	43.1 ± 6.3	43.0 ± 5.9	43.5 ± 7.4	0.63
Mother's age (year)	40.5 ± 4.8	40.7 ± 4.9	40.1 ± 4.8	0.50
Father's body mass index	25.6 ± 4.4	25.9 ± 3.8	25.4 ± 4.0	0.56
Mother's body mass index	24.7 ± 5.0	24.8 ± 4.6	25.4 ± 4.8	0.52

*Note*: Data are expressed as mean ± standard deviation. Data with skewed distributions are expressed as the median with 25th/75th percentiles in parentheses. Skewed data were log‐transformed before statistical analysis.

Abbreviations: HDL, high‐density lipoprotein; LDL, low‐density lipoprotein.

Relative body weight (RBW) and body mass index (BMI)‐Z score were used as the indices of obesity. The RBW (%) was calculated as {(individual body weight)/(age‐, sex‐, and height‐specific body weight from a reference population)–1} × 100. Participants' levels of obesity were defined as mild for those with RBW more than 20% but less than 30%, moderate for those with more than 30% but less than 50%, and severe for those with above 50%. BMI was calculated as (weight in kg)/(height in m).^2^ Some previous studies on the treatment of childhood obesity have reported the results using BMI‐Z scores. To compare the effect of the present study with that of previous studies, BMI‐Z scores were retrospectively calculated using Japanese reference values.^19^ Because participants were in a growth phase, the treatment effect was determined by the change in RBW, taking into account the increase in height. In the treatment of obesity, what is important is not only how much weight was lost but also how much incidence of disease caused by obesity was reduced by weight loss; however, there are no reports showing specific data in children. We therefore referred to previous studies in the area of adult obesity treatment. The Finnish Diabetes Prevention Study reported that the risk of diabetes was reduced by 58% in adult patients with impaired glucose tolerance who obtained approximately 5% weight loss (−4.7% ± 5.4%).^20^ A categorical analysis of weight loss from the Look AHEAD trial showed that improvement was obtained with weight loss of 5% or more for systolic and diastolic blood pressure, HDL‐cholesterol, and triglycerides.^21^ Therefore, the Expert panel by the American Heart Association/the American College of Cardiology/the Obesity Society recommended as an initial goal the loss of 5%–10% of baseline weight.^22^, ^23^

The average RBW reduction was 8.6% for 13 participants who were able to lose 5% (equal to or more than 4.5% but less than 5.5%) of their body weight within a few months. Therefore, we defined successful treatment as a final decrease in RBW of ≥8.6%.

At the first visit, participants and their parents received a 20‐ to 30‐min instruction on how to improve lifestyle; at subsequent visits, they received a 5‐ to 10‐min instruction to intensify lifestyle modification. Lifestyle modification included promoting PA and modifying dietary intake. We recommended walking more than 10,000 steps/day while on holidays^13^ using a pedometer (HJ‐203, Omron, Kyoto, Japan) to promote PA. We asked participants to attempt the following measures to modify dietary intake: (1) chewing each mouthful more than 20 times, (2) drinking water or (green or oolong) tea instead of juice, (3) increasing vegetable intake, and (4) avoiding second helpings. Participants were also asked to fill out a form every day to record whether they had achieved each of the lifestyle modifications. Participants were asked to record the number of steps walked on holidays. We also recommended parents to cooperate with their children and walk with them, particularly if the children were elementary school students. We did not ask participants and their parents to undergo strict calorie restriction, but instructed them to chew well to help feel fuller as a way of reducing their intake.^24^, ^25^ The second outpatient visit was scheduled 2–3 weeks after the first visit to maintain motivation. The third and subsequent visits were scheduled once a month. The data from the beginning of treatment were used because improvement of obesity after 1 year is associated with improvement during the early stage of treatment.^26^

At every visit, levels of the following CV risk factors were examined: height, weight, waist circumference, blood pressure, and blood biochemical parameters. The waist circumference was measured at the level of the navel in a standing position. Blood samples were collected in the morning after an overnight fast. Blood biochemistry values examined were triglycerides, high‐density lipoprotein (HDL) cholesterol, total cholesterol, fasting plasma glucose (FPG), insulin, aspartate aminotransferase, alanine aminotransferase, and uric acid. Blood pressure was measured three times using an automated sphygmomanometer (TM‐2571, A&D, Tokyo, Japan), and the mean values from the last two measurements were used. Levels of HDL‐cholesterol were determined by a direct quantitative assay, and levels of triglycerides, total cholesterol, aspartate aminotransferase, alanine aminotransferase, and uric acid were determined by enzymatic assays using an automated analyzer (JCA‐BM6050; JEOL Ltd., Tokyo, Japan). Levels of FPG were determined by the hexokinase method using an automated analyzer (ADAMS® Glucose GA‐1170, ARKRAY, Kyoto, Japan). Insulin levels were measured by a chemiluminescence immunological assay using an automated analyzer (Lumipulse® PrestoII; Fujirebio Inc., Tokyo, Japan). All serum biochemical parameters were analyzed in the NHO Kagoshima Medical Center. Non‐HDL‐cholesterol was calculated as (total cholesterol) − (HDL‐cholesterol). Furthermore, the number of steps walked on holidays between each visit was also assessed.

Differences in mean values were analyzed using paired or unpaired *t*‐tests as appropriate. In the treatment continuation group, predictors of final RBW reduction were examined, focusing on the level of RBW reduction and the number of holiday steps taken in the early phase of treatment. A univariate regression analysis was performed with the final RBW reduction as the dependent variable. The independent variables were the level of RBW reduction and the number of steps walked on holidays between the previous visit and each subsequent visit in the early stages of treatment (second, third, and fourth visits). Multivariate regression analysis was performed using the parameters that were significant in the univariate regression analysis. To examine the factors in dropout, a logistic regression analysis was performed with dropout or not as the dependent variable. The independent variables were each parameter at the first visit, the level of RBW reduction, and the number of steps walked on holidays between the previous visit and each subsequent visit. Statistical analysis was performed using IBM SPSS® Statistics Version 23.0 (IBM Japan, Ltd., Tokyo, Japan). A two‐tailed probability value of <0.05 was considered statistically significant.

## RESULTS

### Characteristics of final participants

The final number of participants who fulfilled the inclusion criteria was 131 (Table 2). The mean age (± standard deviation) at the first visit was 10.1 (±2.4) years (5–17 years), and there were 74 boys and 57 girls. Regarding the level of obesity, 23 participants (17.6%) were mild, 74 (56.5%) were moderate, and 34 (26.0%) were severe levels of obesity. The mean length of the follow‐up was 1.25 (±1.33) years.

**TABLE 2:** Characteristics of final 131 participants at the first and the final visits.

	First	Final	*p* Value
Age (years)	10.1 ± 2.4	11.4 ± 2.5	<0.001
Sex (M/F)	74/57	74/57	
Height (cm)	140.6 ± 12.5	147.5 ± 12.6	<0.001
Weight (kg)	52.3 ± 16.3	53.5 ± 15.0	0.06
Relative body weight (%)	45.8 ± 20.4	31.0 ± 20.0	<0.001
Level of obesity (normal/mild/moderate/severe)	0/23/74/34	38/30/44/19	
Body mass index	25.8 ± 4.2	24.2 ± 4.0	<0.001
Body mass index‐Z scores	2.2 ± 0.4	1.6 ± 0.6	<0.001
Waist circumference (cm)	83.9 ± 11.7	80.8 ± 11.2	<0.001
Systolic blood pressure (mmHg)	108 ± 10	104 ± 10	<0.001
Diastolic blood pressure (mmHg)	62 ± 8	59 ± 7	<0.001
Aspartate aminotransferase (U/L)	31 ± 19	23 ± 6	<0.001
Alanine aminotransferase (U/L)	42 ± 50	22 ± 13	<0.001
Uric acid (μmol/L)	318.6 ± 79.7	313.3 ± 75.6	0.24
Total cholesterol (mmol/L)	4.61 ± 0.80	4.31 ± 0.82	<0.001
Triglycerides (mmol/L)	1.08 ± 0.50	0.85 ± 0.39	<0.001
HDL‐cholesterol (mmol/L)	1.29 ± 0.27	1.39 ± 0.28	<0.001
LDL‐cholesterol (mmol/L)	2.89 ± 0.75	2.58 ± 0.70	<0.001
Fasting plasma glucose (mmol/L)	4.88 ± 0.46	4.87 ± 0.47	0.92
Insulin (pmol/L)	114.4 ± 63.5	92.4 ± 53.1	<0.001

*Note*: Data are expressed as mean ± standard deviation. Data with skewed distributions are expressed as the median with 25th/75th percentiles in parentheses. Skewed data were log‐transformed before statistical analysis.

Abbreviations: HDL, high‐density lipoprotein; LDL, low‐density lipoprotein.

Comparison of each parameter at the time of the first and the final visits showed a significant improvement in the indices of obesity (the RBW and BMI‐Z score) (Table 2). There was also a substantial improvement in all biochemical measures except for uric acid and FPG level. The mean reductions in RBW and BMI‐Z scores were 14.7% (±12.8%) and 0.64 (±0.48), respectively. The successful treatment rate was 68.0% when a reduction in RBW of 8.6% or more was defined as successful treatment.

### Predictors of final RBW reduction

In the univariate regression analysis, RBW at the start of treatment and the level of RBW reduction from the previous visit at all visits were significant (Table 3). Regarding the number of holiday steps, the number of steps walked on holiday from the second to the third visit (number of holiday steps at the third visit) was significant. Multivariate regression analysis showed that RBW at the first visit and the number of steps walked on holiday from the second to the third visits were independently significant predictors.

**TABLE 3:** Predictors of final relative body weight reduction.

Variable	*N*	Univariate			Multivariate (*N* = 105)		
		*β*	OR (95% CI)	*p* Value	*β*	OR (95% CI)	*p* Value
At the 1st visit							
Age (years)	131	−0.11	−0.51 (−1.49, 0.33)	0.21			
Sex^a^	131	−0.02	−0.39 (−4.89, 4.10)	0.86			
Relative body weight (RBW)	131	−0.35	−0.22 (−0.32, −0.12)	<0.001	−0.21	−0.13 (−0.25, −0.01)	0.03
Father's age	99	0.16	0.33 (−0.09, 0.75)	0.12			
Mather's age	110	0.09	0.24 (−0.25, 0.74)	0.34			
Father's body mass index	84	−0.08	−0.28 (−1.02, 0.45)	0.44			
Mather's body mass index	89	−0.12	−0.33 (−0.92, 0.27)	0.28			
At the early stages of treatment (2nd, 3rd, and 4th visits)							
Decrease in RBW from the 1st to the 2nd visits (%)	131	0.36	1.43 (0.79, 2.07)	<0.001	0.16	0.64 (−0.12, 1.41)	0.10
Mean number of steps on holidays between the 1st and 2nd visits (per 1000 steps)^b^	92	−0.16	−0.60 (−1.37, 0.16)	0.12			
Decrease in RBW from the 2nd to the 3rd visits (%)	131	0.37	1.56 (0.87, 2.25)	<0.001	0.16	0.70 (−0.08, 1.47)	0.08
Mean number of steps on holidays between the 2nd and the 3rd visits (per 1000 steps)^b^	105	−0.27	−1.10 (−1.87, −0.33)	0.006	−0.19	−0.77 (−1.50, −0.05)	0.04
Decrease in RBW from the 3rd to the 4th visits (%)	131	0.34	1.51 (0.80, 2.23)	<0.001	0.14	0.63 (−0.18, 1.43)	0.13
Mean number of steps on holidays between the 3rd and the 4th visits (per 1000 steps)^b^	98	−0.11	−0.50 (−1.40, 0.40)	0.27			

Abbreviations: *β*, standardized partial regression coefficient; CI, confidence interval; OR, odds ratio.

^a^ Statistical analysis was performed with boys = 1 and girls = 2.

^b^ The mean number of steps per day divided by 1000 was used for analysis.

Next, in order to examine the predictive factors after the start of treatment (after the first visit), the analysis was conducted excluding the parameters at the first visit (Table 4). The multivariate regression analysis revealed that the level of RBW reduction between the first and second visits and the number of holiday steps from the second to the third visits were independently significant predictors.

**TABLE 4:** Predictors of final relative body weight reduction after the start of treatment.

Variable	*N*	Univariate			Multivariate (*N* = 105)		
		*β*	OR (95% CI)	*p* Value	*β*	OR (95% CI)	*p* Value
At the early stages of treatment (2nd, 3rd, and 4th visits)							
Decrease in RBW from the 1st to the 2nd visits (%)	131	0.36	1.43 (0.79, 2.07)	<0.001	0.24	0.95 (0.22, 1.68)	0.01
Mean number of steps on holidays between the 1st and 2nd visits (per 1000 steps)^a^	92	−0.16	−0.60 (−1.37, 0.16)	0.12			
Decrease in RBW from the 2nd to the 3rd visits (%)	131	0.37	1.56 (0.87, 2.25)	<0.001	0.18	0.78 (−0.005, 1.56)	0.051
Mean number of steps on holidays between the 2nd and the 3rd visits (per 1000 steps)^a^	105	−0.27	−1.10 (−1.87, −0.33)	0.006	−0.19	−0.79 (−1.52, −0.05)	0.04
Decrease in RBW from the 3rd to the 4th visits (%)	131	0.34	1.51 (0.80, 2.23)	<0.001	0.14	0.62 (−0.20, 1.44)	0.14
Mean number of steps on holidays between the 3rd and the 4th visits (per 1000 steps)^a^	98	−0.11	−0.50 (−1.40, 0.40)	0.27			

Abbreviations: *β*, standardized partial regression coefficient; CI, confidence interval; *N*, number; OR, odds ratio; RBW, relative body weight.

^a^ The mean number of steps per day divided by 1000 was used for analysis.

### Predictors of dropout cases

Among the parameters at the second and third visits, a small number of holiday steps at the second visit (i.e., the number of steps walked on holiday between the first and second visits) predicted dropout (Table 5). The mean number of holiday steps was significantly higher in the treatment continuation group (8246 ± 2933 steps) than in the dropout group (6728 ± 2398, *p* = 0.03).

**TABLE 5:** Predictive factors for dropout.

Variable	*N*	OR (95% CI)	*p* Value
At the 1st visit			
Age (years)	169	1.04 (0.90, 1.20)	0.60
Sex^a^	171	0.63 (0.30, 1.32)	0.22
Relative body weight (RBW)	171	1.01 (0.99, 1.02)	0.59
Body mass index‐*Z* score	147	1.20 (0.51, 2.80)	0.68
Father's age	130	1.02 (0.95, 1.08)	0.63
Mother's age	145	0.97 (0.90, 1.05)	0.50
Father's body mass index	111	0.97 (0.86, 1.09)	0.55
Mather's body mass index	121	1.03 (0.94, 1.12)	0.52
At the early stages of treatment (2nd, 3rd visits)			
Decrease in RBW from the 1st to the 2nd visit (%)	171	1.00 (0.89, 1.11)	0.97
Mean number of steps on holidays between the 1st and the 2nd visits(per 1000 steps)^b^	115	0.83 (0.70, 0.98)	0.03
Decrease in RBW from the 2nd to the 3rd visit (%)	151	1.07 (0.92, 1.25)	0.39
Mean number of steps on holidays between the 2nd and the 3rd visits(per 1000 steps)^b^	112	0.95 (0.73, 1.24)	0.71

Abbreviations: CI, confidence interval; *N*, number; OR, odds ratio.

^a^ Statistical analysis was performed with boys = 1 and girls = 2.

^b^ The mean number of steps per day divided by 1000 was used for analysis.

## DISCUSSION

This study revealed that mean changes in RBW and BMI‐Z scores after following the walking strategy were −14.7% and −0.64%, respectively. Predictive factors for a larger reduction after the initiation of treatment were the level of RBW reduction between the first and the second visits and the number of holiday steps between the second and the third visits. The study also showed that a small number of steps walked on holiday between the first and the second visits were a significant predictive factor for dropout cases.

Major concern of the strategy for treating obesity is its efficacy and feasibility. We focused on promoting walking, which is relatively easy to incorporate into daily life. Mead et al. reviewed 70 randomized controlled trials that had been performed until 2016 to assess the effects of multicomponent intervention (diet, physical activity, and behavioral interventions) for the treatment of children with overweight/obesity aged 6–11 years.^27^ Among these, the mean change in BMI‐Z scores of the intervention groups, but not the mean difference between intervention and control groups, was −0.15 ± 0.16 (95% confidence interval, −0.20 and −0.09; range, −0.71 to +0.1) in 37 trials that used BMI‐Z scores as the index of obesity.^27^ Boutelle et al. reported similar effectiveness of a randomized clinical trial in 2017 where the change in the mean BMI‐Z score of 8‐ to 12‐year‐old children was −0.20 after a 12‐month family‐based treatment program.^28^ Recent reports have shown more efficient data. Siegrist et al. described in 2021 that the change in the mean BMI‐Z score was −0.29 in school‐aged children (14.0 ± 1.8 years) at the time of 1‐year follow‐up after a 4‐week multicomponent intervention.^26^ Fowler et al. reported in 2021 that the change in the mean BMI‐Z score of children aged 7 to 11 years was **−**0.40 from the baseline to the end of 12 months of family‐based treatment program.^29^ The mean decrease in BMI‐Z score in this study (**−**0.64 ± 0.48) was equal to or greater than those in previous studies.^26^, ^27^, ^28^, ^29^

In the present study, participants who were able to reduce RBW in the early phase of treatment showed greater final RBW reduction. It has been reported that short‐term weight loss in the early phase of treatment leads to long‐term weight loss.^26^, ^30^, ^31^ In the long‐term effects of lifestyle intervention, Siegrist et al. reported that even lifestyle interventions of only 4 weeks were effective for weight loss after 1 year.^26^ In a randomized controlled trial of a 20‐week family‐based behavioral weight loss treatment conducted on children with obesity aged 7–12 years, Goldschmidt et al. reported that early weight loss was associated with treatment efficacy at the end of treatment and at 2‐year follow‐up.^32^ Goldschmidt et al. also cited self‐efficacy as an important determinant of the ultimate success of obesity treatment.^32^ They stated that, as behavior change in patients is more likely to occur in the early stages of treatment, it is important that affected children and their families experience a sense of self‐efficacy during this period.^32^ In the present study, we similarly thought that changing lifestyles (through introducing walking and modifying eating behaviors) and experiencing the success of weight loss in the early phase would cause participants to maintain and/or increase their motivation for treating obesity, resulting in a greater final RBW reduction. Furthermore, in this study, the predictor of dropout was a low number of holiday steps from the first to the second visit, suggesting that the inability to develop exercise habits and establish self‐efficacy early in treatment was likely to lead to treatment dropout.

The present study showed a significant improvement in all biochemical indicators between the first and the last visits except for uric acid and FPG levels. The reason why uric acid levels did not change significantly following treatment requires further investigation, but it may reflect the fact that the level of improvement in obesity, as defined in this study, was not sufficient to affect uric acid levels. Additionally, this study only assessed changes in measured values of uric acid and may not have taken into account the increases in uric acid levels with age seen during childhood. Average blood glucose levels prior to treatment were 4.88 mmol/L (88 mg/dL), within normal ranges, which may explain why they remained unchanged after treatment. Insulin levels decreased significantly (*p* < 0.001) from 114.4 ± 63.5 pmol/L to 92.4 ± 53.1 pmol/L following treatment, suggesting that obesity‐related high insulin levels can be improved following walking therapy.

There are some limitations. First, this study focused only on the number of holiday steps and did not evaluate weight loss due to improved diet, although it was our aim to focus on walking, but not on strict diet limitation. Second, the number of holiday steps taken before the start of treatment was not measured and was unconfirmed with regard to the exercise habits of the subjects prior to the start of treatment.

## CONCLUSIONS

In conclusion, **t**he present study showed that the effect of walking was comparable to or greater than that of previous strategies.^27^, ^28^ Predictors of final RBW reduction were the level of RBW reduction from the first to the second visits and the mean number of steps on holiday between the second and the third visits. Fewer steps on holiday between the first and the second visits were a predictor of dropout. These data suggest that walking on holidays is an effective measure to treat childhood obesity and that it is important to help children with obesity develop walking or light‐intensity PA habits and reduce RBW during the early stages of treatment.

## AUTHOR CONTRIBUTIONS

M.Y. contributed to the conception and design of this study. K.M., Y.N., S.S., Y.I., and M.Y. collected and analyzed data. K.M. drafted the manuscript. M.Y. and Y.N. critically reviewed the manuscript and supervised the whole study process. All authors read and approved the final manuscript.

## CONFLICT OF INTEREST STATEMENT

The authors declare no conflict of interest.

## ETHICS STATEMENT

This study was approved by the Ethics Committee of the NHO Kagoshima Medical Center (No. 2021‐20).

## References

[1] Jebeile H , Kelly AS , O'Malley G , Baur LA . Obesity in children and adolescents: epidemiology, causes, assessment, and management. Lancet Diabetes Endocrinol. 2022;10:351–365. 35248172 10.1016/S2213-8587(22)00047-X PMC9831747

[2] World Health Organization . Prevalence of obesity among children and adolescents, BMI > +2 standard deviation above the median, crude Estimates by WHO region. 2023. [cited 2023 Oct 22]. Available from: https://apps.who.int/gho/data/view.main.BMIPLUS2REGv?lang=en

[3] Ministry of Education, Culture, Sports, Science and Technology . Annual Report of School Health Survey of 2022. Tokyo: The Printing Office, Ministry of Finance (in Japanese). 2022. [cited 2023 Oct 22]. Available from: https://www.mext.go.jp/content/20221125‐mxt_chousa01‐000023558.pdf

[4] Kidokoro T , Tomkinson GR , Lang JJ , Suzuki K . Physical fitness before and during the COVID‐19 pandemic: results of annual national physical fitness surveillance among 16,647,699 Japanese children and adolescents between 2013 and 2021. J Sport Health Sci. 2023;12(2):246–254. 36343895 10.1016/j.jshs.2022.11.002 PMC9635948

[5] Yoshinaga M . Childhood obesity and problems. J Japan Med Assoc. 2016;145:543–545. (in Japanese).

[6] Pulgarón ER . Childhood obesity: a review of increased risk for physical and psychological comorbidities. Clin Ther. 2013;35:A18–A32. 23328273 10.1016/j.clinthera.2012.12.014 PMC3645868

[7] Horesh A , Tsur AM , Bardugo A , Twig G . Adolescent and childhood obesity and excess morbidity and mortality in young adulthood – a systematic review. Curr Obes Rep. 2021;10:301–310. 33950400 10.1007/s13679-021-00439-9

[8] Jebeile H , Cardel MI , Kyle TK , Jastreboff AM . Addressing psychosocial health in the treatment and care of adolescents with obesity. Obesity (Silver Spring). 2021;29:1413–1422. 34431234 10.1002/oby.23194

[9] Barlow SE , Expert Committee . Expert committee recommendations regarding the prevention, assessment, and treatment of child and adolescent overweight and obesity: summary report. Pediatrics. 2007;120(Suppl 4):S164–S192. 18055651 10.1542/peds.2007-2329C

[10] Steinbeck KS , Lister NB , Gow ML , Baur LA . Treatment of adolescent obesity. Nat Rev Endocrinol. 2018;14:331–344. 29654249 10.1038/s41574-018-0002-8

[11] Kahlmeier S , Wijnhoven TM , Alpiger P , Schweizer C , Breda J , Martin BW . National physical activity recommendations: systematic overview and analysis of the situation in European countries. BMC Public Health. 2015;15:133. 25879680 10.1186/s12889-015-1412-3 PMC4404650

[12] Hallal PC , Andersen LB , Bull FC , Guthold R , Haskell W , Ekelund U , et al. Global physical activity levels: surveillance progress, pitfalls, and prospects. Lancet. 2012;380(9838):247–257. 10.1016/S0140-6736(12)60646-1 22818937

[13] Smith L , Ekelund U , Hamer M . The potential yield of non‐exercise physical activity energy expenditure in public health. Sports Med. 2015;45:449–452. 25648364 10.1007/s40279-015-0310-2

[14] Herzig KH , Ahola R , Leppäluoto J , Jokelainen J , Jämsä T , Keinänen‐Kiukaanniemi S . Light physical activity determined by a motion sensor decreases insulin resistance, improves lipid homeostasis and reduces visceral fat in high‐risk subjects: PreDiabEx study RCT. Int J Obes (Lond). 2014;38:1089–1096. 24285336 10.1038/ijo.2013.224 PMC4125749

[15] Maturi MS , Afshary P , Abedi P . Effect of physical activity intervention based on a pedometer on physical activity level and anthropometric measures after childbirth: a randomized controlled trial. BMC Pregnancy Childbirth. 2011;11:103. 10.1186/1471-2393-11-103 22176722 PMC3292461

[16] Carlin A , Murphy MH , Gallagher AM . Do interventions to increase walking work? A systematic review of interventions in children and adolescents. Sports Med. 2016;46:515–530. 26626069 10.1007/s40279-015-0432-6 PMC4801983

[17] Mitsui T , Barajima T , Kanachi M , Shimaoka K . The significant drop in physical activity among children on holidays in a small town in the Tohoku district. J Physiol Anthropol. 2010;29:59–64. 20551585 10.2114/jpa2.29.59

[18] Yoshinaga M , Miyazaki A , Aoki M , Ogata H , Ito Y , Hamajima T , et al. Promoting physical activity through walking to treat childhood obesity, mainly for mild to moderate obesity. Pediatr Int. 2020;62:976–984. 32304151 10.1111/ped.14253

[19] Kato N , Takimoto H , Sudo N . The cubic function for spline smoothed L, S and M values for BMI reference data of Japanese children. Clin Pediatr Endocrinol. 2011;20:47–49. 23926394 10.1297/cpe.20.47 PMC3687634

[20] Tuomilehto J , Lindstrom J , Eriksson JG , Valle TT , Hämäläinen H , Ilanne‐Parikka P , et al. Prevention of type 2 diabetes mellitus by changes in lifestyle among subjects with impaired glucose tolerance. N Engl J Med. 2001;344:1343–1350. 11333990 10.1056/NEJM200105033441801

[21] Wing RR , Lang W , Wadden TA , Safford M , Knowler WC , Bertoni AG , et al. Benefits of modest weight loss in improving cardiovascular risk factors in overweight and obese individuals with type 2 diabetes. Diabetes Care. 2011;34:1481–1486. 21593294 10.2337/dc10-2415 PMC3120182

[22] Jensen MD , Ryan DH , Apovian CM , Ard JD , Comuzzie AG , Donato KA , et al. 2013 AHA/ACC/TOS guideline for the management of overweight and obesity in adults: a report of the American College of Cardiology/American Heart Association Task Force on Practice Guidelines and the Obesity Society. Circulation. 2014;129(25 Suppl 2):S102–S138. 24222017 10.1161/01.cir.0000437739.71477.ee PMC5819889

[23] Williamson DA , Bray GA , Ryan DH . Is 5% weight loss a satisfactory criterion to define clinically significant weight loss? Obesity (Silver Spring). 2015;23:2319–2320. 26523739 10.1002/oby.21358

[24] Miquel‐Kergoat S , Azais‐Braesco V , Burton‐Freeman B , Hetherington MM . Effects of chewing on appetite, food intake and gut hormones: a systematic review and meta‐analysis. Physiol Behav. 2015;1(151):88–96. 10.1016/j.physbeh.2015.07.017 26188140

[25] Yoshimatsu H , Itateyama E , Kondou S , Tajima D , Himeno K , Hidaka S , et al. Hypothalamic neuronal histamine as a target of leptin in feeding behavior. Diabetes. 1999;48:2286–2291. 10580415 10.2337/diabetes.48.12.2286

[26] Siegrist M , Heitkamp M , Braun I , Vogg N , Haller B , Langhof H , et al. Changes of omentin‐1 and chemerin during 4 weeks of lifestyle intervention and 1 year follow‐up in children with obesity. Clin Nutr. 2021;40:5648–5654. 34666255 10.1016/j.clnu.2021.09.042

[27] Mead E , Brown T , Rees K , Azevedo LB , Whittaker V , Jones D , et al. Diet, physical activity and behavioural interventions for the treatment of overweight or obese children from the age of 6 to 11 years. Cochrane Database Syst Rev. 2017;6(6):CD012651. 10.1002/14651858.CD012651 28639319 PMC6481885

[28] Boutelle KN , Rhee KE , Liang J , Braden A , Douglas J , Strong D , et al. Effect of attendance of the child on body weight, energy intake, and physical activity in childhood obesity treatment: a randomized clinical trial. JAMA Pediatr. 2017;171:622–628. 28558104 10.1001/jamapediatrics.2017.0651 PMC5710344

[29] Fowler LA , Grammer AC , Ray MK , Balantekin KN , Stein RI , Kolko Conlon RP , et al. Examining the interdependence of parent‐child dyads: effects on weight loss and maintenance. Pediatr Obes. 2021;16:e12697. 32720457 10.1111/ijpo.12697 PMC8186411

[30] Gow ML , Baur LA , Ho M , Chisholm K , Noakes M , Cowell CT , et al. Can early weight loss, eating behaviors and socioeconomic factors predict successful weight loss at 12‐ and 24‐months in adolescents with obesity and insulin resistance participating in a randomised controlled trial? Int J Behav Nutr Phys Act. 2016;13(1):43. 10.1186/s12966-016-0367-9 27036113 PMC4818484

[31] Martos‐Moreno GÁ , Martínez‐Villanueva Fernández J , Frías‐Herrero A , Martín‐Rivada Á , Argente J . Conservative treatment for childhood and adolescent obesity: real world follow‐up profiling and clinical evolution in 1300 patients. Nutrients. 2021;28(13):3847. 10.3390/nu13113847 PMC8624087 34836102

[32] Goldschmidt AB , Stein RI , Saelens BE , Theim KR , Epstein LH , Wilfley DE . Importance of early weight change in a pediatric weight management trial. Pediatrics. 2011;128:e33–e39. 21690118 10.1542/peds.2010-2814 PMC3124104

